# Coronavirus vaccine hesitancy among unvaccinated Austrians: Assessing underlying motivations and the effectiveness of interventions based on a cross-sectional survey with two embedded conjoint experiments

**DOI:** 10.1016/j.lanepe.2022.100389

**Published:** 2022-04-22

**Authors:** Tanja A. Stamm, Julia Partheymüller, Erika Mosor, Valentin Ritschl, Sylvia Kritzinger, Jakob-Moritz Eberl

**Affiliations:** a Institute for Outcomes Research, Center for Medical Statistics, Informatics and Intelligent Systems, Medical University of Vienna, Spitalgasse 23, Vienna 1090, Austria; b Ludwig Boltzmann Institute for Arthritis and Rehabilitation, Vienna, Austria; c Department of Government, University of Vienna, Austria; d Department of Communication, University of Vienna, Vienna, Austria; e Department of Media and Communication, Ludwig Maximilian University of Munich, Germany

**Keywords:** Coronavirus, Vaccine hesitancy, Interventions, Conjoint experiment, Cross-sectional survey, COVID-19, Coronavirus Disease 2019

## Abstract

**Background:**

To date, Austria is among the countries with the lowest coronavirus vaccination rates in Western Europe. It has announced the introduction of a general vaccine mandate but is experiencing an increasing societal polarization over this issue. We, therefore, aimed to provide evidence on the underlying motivations of vaccine hesitancy and evaluate what kinds of interventions – information, incentives, and rules – might increase vaccination readiness.

**Method:**

We conducted a cross-sectional survey with a sample of 1,543 unvaccinated Austrian residents in October 2021, including two embedded conjoint experiments.

**Findings:**

We screened 8,190 individuals to recruit the sample matching the Austrian micro-census. In experiment 1, easing rather than tightening of societal restrictions, a fixed monetary reward compared to a lottery and physicians’ recommendations were associated with significantly higher intentions to get vaccinated. In experiment 2, standard approval by European or national authorities and simple information had a significant positive effect on vaccination propensity. Among the unvaccinated, fear of side effects, beliefs that comorbidities or the desire to have children would not allow vaccination, the assumption that the own immune system would provide sufficient protection, conspirational thinking (e.g., the refusal to participate in a ‘large genetic experiment’), low trust in societal institutions, and spiritual beliefs were very common.

**Interpretation:**

While many unvaccinated showed a low propensity to become vaccinated, we identified a cluster of 195 (23% of the participants without missing values) that could potentially be reached by information and incentives, including people with heightened comorbidity rates or a desire for children.

**Funding:**

Vienna Science and Technology Fund

**Research in context**

**Evidence before this study**

Assessments of the effectiveness of interventions in countries like Austria with high COVID-19 vaccine hesitancy paired with increasing societal polarization are currently still rare. We searched medical and social science databases including Web of Science, PubMed, the preprint medRxiv server, and Google Scholar with the terms ‘conjoint experiment’, ‘COVID-19 vaccination’, ‘COVID-19 vaccine hesitancy’, ‘representative’, and ‘Austria’. Until Dec, 15^th^ 2021, we did not find population-representative conjoint experiments with unvaccinated individuals that were conducted in Austria. Moreover, although vaccination campaigns were rolled out in Western Europe in 2021, vaccination rates in many countries plateaued long before reaching their target level. To our knowledge, few studies provide sufficient policy guidance to design suitable interventions to overcome vaccine hesitancy at a stage where some of the measures identified in previous research have already been implemented but were not yet successful.

**Added value of this study**

This study provides evidence on the underlying motivations of vaccine hesitancy. It assesses preferences for different bundles of measures and their effect on the readiness to become vaccinated based on a cross-sectional survey of 1,543 unvaccinated Austrian residents at a point in vaccine rollout when vaccination rates have stagnated.

**Implications of all the available evidence**

Easing societal restrictions where possible, offering a fixed monetary reward as an incentive, taking the necessary steps to reach standard marketing authorization, involving physicians in the vaccination campaign, and focusing on vaccine effectiveness while communicating risks clearly and transparently are recommended as measures to reduce vaccine hesitancy. Reaching out to unvaccinated people with comorbidities and those in the child-bearing age should be a priority.

Alt-text: Unlabelled box

## Introduction

In many countries, flattening vaccination curves currently indicate high levels of Coronavirus Disease 2019 (COVID-19) vaccine hesitancy. In addition to other non-pharmaceutical measures that can also provide partial protection, adequate vaccinations can rates help to prevent severe and fatal courses of COVID-19, in a timely manner. After an initial shortage of vaccine supply, many of the most developed nations are now facing a shortage of demand. Even though vaccinations are now widely available to everyone in those countries and offered at a low-threshold level, many citizens have remained unvaccinated.^1^

During several stages of the global vaccine roll-out, the German-speaking countries showed the lowest COVID-19 vaccination rates in Western Europe, with Austria having long held the highest share of unvaccinated people among these countries.^1^, ^2^, ^3^ In February 2022, Austria stands at a rate of 73.2% of people who received at least two doses. Western European countries with lower rates include Netherlands (70.8%), Greece (69.9%), Luxemburg (68.5%) and Cyprus (53.4%).^4^ To increase the vaccination rate, the Austrian government has announced the introduction of a general COVID-19 vaccine mandate but is experiencing increasing societal polarization over this issue.^5^ Public discourse has often focused on the actions of the most extreme group of vaccination opponents (e.g. demonstrations, forging vaccination certificates, de-registrations from the public health registry). However, population-representative survey data for Austria reveal that vaccine hesitancy is a matter of degree, with some of the unvaccinated being more opposed to vaccinations than others,^6^ and that vaccine attitudes have changed considerably over time.^7^ Hence, at least a fraction of the remaining unvaccinated population might still be reached and decide to become vaccinated.

Further research, however, is needed to identify the underlying reasons for vaccine hesitancy of the remaining unvaccinated and to provide guidance on how to design appropriate interventions to reach them specifically. So-called conjoint experiments^8^ seem particularly suitable for this purpose as they allow individuals to compare multiple attributes simultaneously and rate them concerning the likelihood to influence the decision to vaccinate. Earlier conjoint experiments on COVID-19 vaccine acceptance have mainly focused on preferences regarding the features of vaccines.^9^, ^10^, ^11^, ^12^, ^13^, ^14^ Yet, characteristics of vaccines and facts about a disease are usually given and cannot be modified in the short run. Instead, communication – possibly paired with incentives – has the potential to reduce fears and ensure broader acceptance. A study in Germany from March 2021 has investigated specific policies, such as financial incentives, granting freedoms, and the vaccination at local doctors,^15^ finding moderate positive effects on the vaccination rate of these measures. Information provided in the mother tongue for migrants,^16^ given by different health professionals,^17^ targeting patients with certain disease or specifically health professionals themselves^18^ have been tried out in other countries. Some of these interventions, such as information in different languages, vaccinations at general practitioners and vaccine passports granting freedoms have already been implemented in Austria. The latter led to increased vaccination readiness in neighboring countries.^19^ Still, it remains unclear what other measures can be effective to reach the group of unvaccinated that has remained persistent even after a majority of the Austrian population was vaccinated already. Existing studies do not offer sufficient guidance for designing effective interventions in the current situation, and there is a lack of evidence for effective communication and campaign design in countries such as Austria.

Against this background, we aim to provide evidence on the underlying motivations of vaccine hesitancy and evaluate possible interventions' role. For this purpose, we conducted a cross-sectional survey with a group of 1,543 unvaccinated individuals living in Austria, capturing their preferences, motivations, and concerns regarding COVID-19 vaccination. The survey included two conjoint experiments, with the first evaluating features of a hypothetical vaccination campaign and the second one assessing the role of media coverage.

## Methods

### Participants and survey design

To recruit the sample of unvaccinated participants, we screened a sample of Austrian residents which demographics matching the Austrian micro-census with respect to gender, age group, region of residence, and educational level. As official vaccination statistics suggested that the unvaccinated were predominantly found among younger age cohorts due to the initial lower prioritization regarding vaccination of this group of the population, we included a higher proportion of people up to 39 years (55% of the total sample). Higher vaccination coverage of the overall population, including younger people, could also better protect vulnerable older persons who might, for example, for medical reasons have not been vaccinated. Where sampling did not match the census statistics regarding the gender distribution or the region of residence, we adjusted the scores using population-based weights.

We included participants of 14 to 75 years of age, since from the age of 14, children in Austria can decide for themselves, without their parents, whether they want to be vaccinated. The survey including two embedded conjoint experiments was run between October 6^th^ and 13^th^, 2021. Participants completed a self-administered online questionnaire with sociodemographic information and personal preferences. Details on the survey questions are described in Supplemental File 1. Before the questionnaire was programmed, it was pilot tested by experts as well as lay-persons and adapted based on the feedback received.

We commissioned Marketagent GmbH (https://www.marketagent.com/) to conduct the fieldwork using its pool of respondents and its online access panel of more than 135,000 Austrian residents in total. Marketagent contacted potential participants stratified for gender, age group, region of residence, and educational level to match the Austrian micro-census as closest as possible, asked them about their vaccination status and invited only the unvaccinated participants to complete the survey. Marketagent followed their usual approved consent procedures and is certified under ISO 20252. As the two universities received only fully anonymized data, approval from a Medical Ethical Committee was not required.

### Conjoint experiments

Embedded in the survey, we ran two conjoint experiments following the Hainmueller design.^20^ By varying the attributes’ levels randomly, this design allows identifying which components of a multidimensional treatment are influential. The first experiment focused on a hypothetical vaccination campaign and included the following four attributes: (1) differently worded calls for vaccination (no specific reason given; to protect oneself; to protect others; to return to normality), (2) information on who recommended the vaccination (the Federal Government; a physician; a celebrity whom the participant identified as sympathetic in a previous question), (3) incentives (the vaccination is free of charge; a vaccination lottery; monetary reward of 100€) and (4) societal restrictions depending on vaccination status (lifting all restrictions; access to gastronomy, culture, and leisure activities only for people who are vaccinated or recovered, termed the ‘2G rule’; or access to these places also for unvaccinated individuals with a negative COVID-19 test, termed the ‘3G rule’, which was the status quo at the time of the fieldwork). We present more details on experiment 1 in Supplemental Table 1.

The second experiment dealt with a news report on a hypothetical new vaccine against a novel fictional virus similar to the coronavirus and included three attributes: different ways of presenting information (1) about the effectiveness (absolute and relative frequencies of breakthrough infections; 90% effectiveness explained in simple numbers; absolute numbers of breakthrough infections only), (2) about the risk of side effects (vague verbal information that side effects can occur; verbal information that mild side effects are frequent and severe side effects are rare; verbal and numeric information on the frequency of mild and severe side effects; the latter plus an infographic visualizing the numeric information using a waffle chart) and (3) about the authorization procedure (conditional marketing authorization at the EU level; standard marketing authorization at EU level; standard marketing authorization in Austria) of the new hypothetical vaccine. Details on experiment 2 are included in Supplemental Table 2.

The selection and design of the statements in experiment 1 were based on a review of the interventions used in previous conjoint experiments.^9^, ^10^, ^11^, ^12^, ^13^, ^14^ We then adapted potential statements to the public discourse at the time when our survey was conducted. We formulated the statements for experiment 1 according to actual calls for vaccination, the current rules and societal restrictions as well as communications on easing of restrictions in the way they were used or planned at the time of our survey. This also included statements that promoted a sense of community, e.g. the protection of others - a factor that was identified as a potential facilitator for vaccine readiness in a previous Austrian study.^6^ The vaccine being ’free of charge’ reflected also the current situation and was used as control. The statements in experiment 2 and the infographic were based on the actual current numbers of COVID-19 cases, vaccination rates, breakthroughs, information on side effects in recent or planned campaigns at that time as well as on political discussions on how information on admission procedures of vaccines should be communicated. All statements and the infographic were designed together with communications experts.

Levels were randomly assigned with equal probabilities and repeatedly recombined, with all combinations being plausible and realistic. None of the statements or incentives tested in our experiments was selected because of our personal preferences. We presented the participants two vignettes at the time and repeated this procedure four times overall (two times per experiment). We first asked the participants which of the two shown vignettes would appeal more to them (binary choice); second, the participants rated their vaccination readiness on a scale from 0 (none) to 10 (high) for each vignette separately.

### Statistical methods

Different statistical methods were used at the various stages of the analysis. We describe the initial sample size calculation in Supplemental File 2. We used descriptive statistics for the sociodemographic variables and personal preferences. Population size by gender and number of inhabitants per region were obtained from ‘*Statistik Austria*’^21^ and used to adjust data to match the Austrian micro-census, where necessary. Beliefs of women and men were compared using Chi-Square tests with population-adjusted values. Significance levels were corrected for multiple testing.

Next, to estimate the relative impact of attributes and levels in the conjoint experiments, we computed Average Marginal Component Effects (AMCEs) with 95% Confidence Intervals (CIs) for the preferred choices and the ratings. Relying on both kinds of measures – preferences and behavioral intentions – is particularly relevant to assess the effects of vaccine communication as information ‘treatments’ typically constitute a softer, yet more flexible, kind of intervention than legal rules, which sometimes cannot be modified for various reasons. We present the AMCEs, alongside confidence intervals, in the form of coefficient plots for ease of interpretation (for the full estimation results, Supplemental Tables 3 and 4).

We conducted a qualitative meaning condensation analysis^22^ supported by the Latent Dirichlet Allocation (LDA)^23^ to analyse the answers to the open-ended question on subjective reasons for not being vaccinated. LDA is an unsupervised probabilistic topic modeling technique that extracts the meanings of a pre-defined number of topics. We first divided the participants' answers into meaning units and assigned a code that best represented the meaning of each unit. We then grouped these codes under higher-level topics. In parallel, we created a semantic space of the answers to the open question, stemmed the words, removed stop-words, cast text into a lower case only, removed punctuations, and ran LDA. We then compared the meaning of the topics from both procedures, discussed similarities and differences, and finally decided on the meaning of the topics based on consensus between the researchers.

Finally, to identify relevant subgroups in the population that a vaccination campaign might still reach, we clustered the participants using the unsupervised K-means algorithm,^24^ into distinct, least overlapping subgroups where each participant belonged to only one of them. For this purpose, we calculated overall vaccine hesitancy based on the total sum scores of all case vignette ratings. We also used other variables of which we assumed from the previous analyses that they would best differentiate between subgroups (e.g., gender, education, comorbidities, desire to have children, fear of side effects, trust in science, belief in homeopathy, political preference). We normalized the scales prior to this analysis and removed participants with missing values in the target variables. We summarized how we handled missing values in Supplemental File 3. We used R (https://www.r-project.org/) for the statistical analysis. For reporting, we adhered to the STrengthening the Reporting of OBservational studies in Epidemiology (STROBE) Checklist (Supplemental File 4).

### Role of the funding source

The sponsor had no influence on the study design, the data analysis, the interpretation of the results and the writing of the manuscript.

### Data statement

The dataset and R code can be obtained from the corresponding author upon reasoned request. Since the dataset also contains open, qualitative statements of the participants, we were advised against freely depositing the data to avoid misuse that could lead to further polarization.

## Results

### Sample characteristics

We contacted 8,190 individuals to reach the planned sample size; of these, 6,647 were vaccinated and therefore not asked to complete the survey (Table 1). The remaining 1,543 unvaccinated Austrian residents participated in the survey. Although more males were included in the initial screening, a larger proportion of the unvaccinated sample was female (56%). Age group distribution showed the effect of the young oversampling: the largest age group was the group of 30- to 39-year-olds (24% of the unvaccinated sample). Regional representation matched with the target distributions. As we wanted our results to represent the perspectives of the unvaccinated, we only adjusted scores for the gender distribution of the total Austrian population (51% female) when comparing beliefs of women and men.

**Table 1:** Sample characteristics. Population size by gender and numbers of inhabitants per region were obtained from *Statistik Austria.*^21^ Only the unvaccinated individuals were asked to complete the survey.

	Total sample	Vaccinated	Unvaccinated
Total Austrian population in January 2021 n=8,932,664	8,190	6,647	1,543
Gender (number of inhabitants; % of total population)			
Male (4,396,952; 49.2%)	4,267 (52%)	3,589 (54%)	678 (44%)
Female (4,535,712; 50.8%)	3,902 (48%)	3,039 (46%)	863 (56%)
Other (not reported)	21 (0%)	19 (0%)	1 (0%)
Mean age across all age groups (in years ± SD)	48 ± 16	49 ± 16	44 ± 15
Age groups n (%)	251 (3%)	195 (3%)	56 (4%)
14-19	932 (11%)	675 (19%)	257 (17%)
20-29	1,447 (18%)	1,084 (16%)	363 (24%)
30-39	1,459 (18%)	1,148 (17%)	311 (20%)
40-49	1,869 (23%)	1,561 (23%)	308 (20%)
50-59	1,505 (18%)	1,313 (20%)	192 (12%)
60-69	651 (8%)	603 (9%)	48 (3%)
70-79	72 (1%)	65 (1%)	7 (0%)
80-89	4 (0%)	3 (0%)	1 (0%)
≥89			
Educational status n (%)			
Compulsory school	1,418 (17%)	1,101(13%)	317 (21%)
Apprenticeship, vocational school	3,053 (37%)	2,404 (36%)	649 (42%)
High school	2,236(27%)	1,856(28%)	380(25%)
University education	1,392(17%)	1,219(18%)	173(11%)
Other school type	64 (1%)	48 (1%)	16 (1%)
Do not want to disclose	27 (0%)	19 (0%)	8 (0%)
Region (number of inhabitants; % of total population)			
Vorarlberg (399,237; 4%)	209 (3%)	163 (2%)	46 (3%)
Tyrol (760,101; 9%)	479 (6%)	390 (6%)	89 (6%)
Salzburg (560,710; 6%)	360 (4%)	277 (4%)	83 (5%)
Styria (1,247,077; 14%)	1,126 (14%)	895 (13%)	231 (15%)
Carinthia (562,089; 6%)	465 (6%)	364 (5%)	101 (7%)
Upper Austria (1,495,608; 17%)	1,154 (14%)	867 (13%)	287 (19%)
Lower Austria (1,690,879; 19%)	1,802 (22%)	1,482 (22%)	320 (21%)
Vienna (1,920,949; 22%)	2,271 (28%)	1,941 (29%)	330 (21%)
Burgenland (296,010; 3%)	324 (4%)	268 (4%)	56 (4%)

Two-thirds of the participants (924; 60%) were employed or self-employed; of these, 9% (83) were health professionals, and 4% (40) worked as school or kindergarten teachers. The mean number of people living in the same household was 2.6 ± 1.5; the most frequent ticked range of household net income category was 2,200 to <2,700 € (165; 11%). Four percent of the female participants (31 out of 863) stated that they were pregnant at the survey time. Twenty-four percent (377; 223 women and 154 men) desired to have children in the future. The most frequently ticked category regarding coronavirus testing was more than four times in the past four weeks (659; 43%). The mean number of organ systems affected by comorbidities was 1.2 (±1.0).

Thirteen percent of the participants (197) said that they had been previously diagnosed with COVID-19. Nineteen percent (287) indicated that they had contacted a person who needed hospital care because of a COVID-19 infection. Slightly more than 83% (1,283) scored their overall health status as very good or good. Sixty-four percent of the participants (982) ranked the personal threat from a coronavirus infection for their health as ‘small’ or ‘very small’. Forty-one percent (636) indicated that they would vote either for the *‘Freedom Party of Austria (FPÖ)’* (23%) or ‘*People, Freedom, Fundamental Rights (MFG)’* (18%) if national council elections would take place on the coming weekend. Both political parties are sceptic towards measures implemented by the Austrian government to contain the pandemic and are also against the (later) announced vaccine mandate. Another 40% (614) indicated that they would either vote invalid nor did not want to specify.

### Conjoint experiments 1 and 2

**Figure 1:**
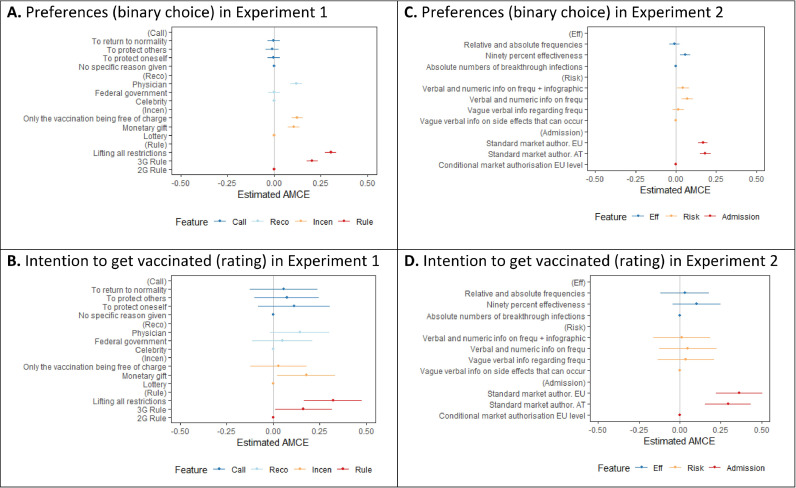
Average Marginal Component Effects (AMCEs) from both experiments for preferences and the intention to get vaccinated. In experiment 1, *(Call)* refers to differently worded calls, *(Reco)* to who recommended the vaccination, *(Incen)* to incentives, and *(Rule)* to societal restrictions. In experiment 2, *(Eff)* refers to effectiveness, *(Risk)* to risk of side effects, and *(Admission)* to market authorization.

The results of the conjoint experiments are shown in Figure 1. In experiment 1, both in terms of preference for a vignette and the behavioral intention to become vaccinated, the strongest effects were observed for legal rules modifications (Figure 1 – A and B). Specifically, we found that the unvaccinated most strongly preferred scenarios that imply fewer societal restrictions for them, and they also reported higher levels of vaccination readiness under these conditions. Incentives and ‘information interventions’ (e.g., reasons to become vaccinated and recommending source) showed weaker effects. Among the incentives, offering a fixed monetary reward had a more positive effect than a vaccination lottery on both preferences and vaccination readiness. Offering the vaccine free of charge was also preferred, but with no significant impact on behavioral intentions. Regarding vaccine communication, the unvaccinated showed a significant preference for physicians’ recommendations compared to those by the Federal Government or celebrities. Messages with reasons to get vaccinated showed no significant effect on either outcome measure.

In experiment 2, standard marketing authorization stood out as a desirable feature in terms of preferences and behavioral intentions (Figure 1 – C and D), with little difference in whether European or national authorities provided such an authorization. Vaccine communication regarding effectiveness and side effects only showed significant effects on preferences, but not on behavioral intentions. News reports focusing on vaccine's effectiveness had a significant positive impact on preferences, compared to news stories about breakthrough infections, even when the reported number of breakthrough infections was chosen to reflect the same rate of effectiveness and the vaccination rate in the population. This suggests that framing vaccine effectiveness in terms of breakthrough infections can undermine confidence in the vaccine, even when the objective effectiveness is the same.

Interestingly, regarding side effects, we found that both combinations of verbal and numeric information, with and without the infographic, appeared more appealing to respondents than vague information. While adding an infographic was not harmful, it did not deliver an additional benefit. Thus, a conventional combination of verbal and numeric information seems to be the most effective way to communicate the frequency of possible side effects transparently and clearly.

In experiments 1 and 2, the ratings of the vignettes for vaccination readiness were low with means of 1.8 (±2.6 and 2.5, respectively) on a 0 to 10 scale. About 43% (669) of all participants rated their willingness to become vaccinated with zero, the lowest score, under all shown scenarios, suggesting that a core of unvaccinated people is very strongly opposed to vaccination. As a result, all effect sizes reported are modest, with a maximum of about 0.25 scale points, for the most impactful interventions. Thus, by changing any individual attribute, we expect that vaccination rates would not increase by more than about 2.5 percentage points. Likewise, shifts in preferences, where respondents were forced to choose one option, were overall modest.

### Underlying attitudes of vaccine hesitancy

**Figure 2:**
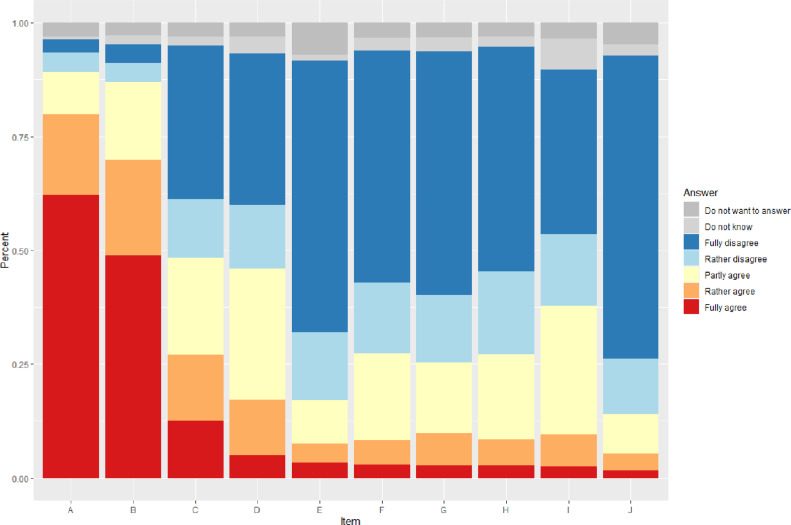
Distributions of responses for an item battery capturing common motivations to become vaccinated (or not). Items are sorted regarding full agreement (in descending order). A majority of the participants agreed or rather agreed that they were concerned about unforeseen side effects of the vaccination (Statement A) and that they would rather prefer to rely on their immune system than on vaccination (B). In contrast, only few participants thought that they were informed enough on how vaccines work (C), that vaccines were helpful for self-protection (D) or the protection of others (F), that vaccines would allow them to live as they did before the pandemic (G), that authorities provided sufficient information about how the vaccines would work (H), that vaccines were safe if the authorities approved them (I), and that they would get vaccinated if others get vaccinated first (J). Lack of time was also no important reason for not getting vaccinated (E).

Figure 2 shows the distributions of responses for an item battery capturing common motivations to become vaccinated (or not). Eighty percent of the participants (1,323) agreed or somewhat agreed that they were concerned about unforeseen side effects of the vaccination, and 70% (1,078) would rather rely on their immune system than on vaccination. In contrast, few participants felt adequately informed about how vaccines work and how they could help protect themselves and others.

Table 2 depicts the spiritual beliefs among respondents. Large proportions of the participants believed in God, life after death, homeopathy, miracles, astrology, and fate which was significantly more common in women than men. In contrast, participants indicated low trust in political and societal institutions, with the government having the lowest (median value of 0 with an interquartile range of 0–3 on a scale from 0 to 10), followed by the pharma industry (1 [0–3]), the media, and the parliament (both 1 [0–4]). More people trusted the healthcare system (4 [1–6]) and even more science (5 [2–6]). No gender differences were seen in the trust scores in institutions.

**Table 2:** Spiritual beliefs among the unvaccinated study participants. The participants could choose between six response options ‘yes’, ‘rather yes’, ‘rather no’, ‘no’, “I do not want to answer” or “I do not know”. In this table, ‘yes’ and ‘rather yes’ were collapsed and the number of women who scored ‘yes’ and ‘rather yes’ were compared to men using Chi Square tests using the population-adjusted values (the p-value in the last columns refers to these tests; the Bonferroni corrected significance level is 0.0083). Of both sexes, 181 (12%) participants indicated that they did not know or did not want to answer if they were to believe in God, 264 (17%) in life after death, 184 (12%) in homeopathy, 147 (10%) in miracles, 161 (10%) in astrology and 119 (8%) in fate.

Belief in…	Men (n)	Women (n)	Men (n) population adjusted	Women (n) population adjusted	Men (%)	Women (%)	p-value
God	333	494	338.3	486.3	56	65	**0.0008**
Life after death	298	525	302.8	516.9	54	73	**<0.001**
Homeopathy	325	561	330.2	552.3	54	74	**<0.001**
Miracles	267	491	271.3	483.4	44	63	**<0.001**
Astrology	177	380	179.8	374.1	29	50	**<0.001**
Fate	398	630	404.4	620.2	63	79	**<0.001**

### Insights from the open-ended question

We received 391 answers (25% of the participants) on potential reasons for not being vaccinated. We extracted four topics: (1) Some people considered themselves healthy with an intact immune system and without the need for a COVID-19 vaccination. (2) Some participants gave a medical condition to why they could not be vaccinated. Examples were chronic diseases (rheumatic diseases, diabetes, multiple sclerosis), stroke, cancer, allergies, alleged earlier vaccination damages, or needle phobia. (3) The third topic referred to the vaccines not being properly developed and authorized. Some responses in this category referred to conspirational thoughts (e.g., *‘a large genetic experiment’* in which they refused to participate), or other concerns related to vaccine development (e.g., the vaccine is produced using *‘genetic engineering’* which they rejected in general). Other participants mentioned an *‘emergency use authorization’* as proof that the vaccines were not sufficiently tested. (4) In the fourth topic, we summarized answers relating to the fear of side effects. Original quotes for each topic are depicted in Table 3.

**Table 3:** Quotes from the answers to the open question. The numbers after the quotes are the participant identification numbers. It was not mandatory to answer the open question and we received answers from 391 participants (25%) on potential reasons for not being vaccinated.

**Healthy with an intact immune system** *‘I feel healthy and am not afraid of a possible infection, because the virus only leads to a respiratory illness in connection with a previous illness, which, such as any stronger flu, can lead to death.’* Participant #1219 *‘I am and remain healthy; and for this I do not need gene therapy.’* Participant #588 *‘I am healthy, move daily in the fresh air, and you can get corona with or without vaccination, with a mild or severe course, and from my friends I know that also vaccinated people die of Corona. So, I see no reason; I remain positive and do not get vaccinated.’* Participant #223
**Belief that a certain medical condition would be a reason to refuse the COVID-19 vaccination** *‘I have had a brain hemorrhage, and I would be dying if I would have a brain hemorrhage again!’* Participant #1110 *‘I had a stroke in 2016, and I do not trust vaccines!’* Participant #244 *‘I am a cancer patient, and there is no information about interactions with all my medications to take.’* Participant #865 *‘According to my doctor, no vaccination is good for me!’* Participant #1069
**Insufficient development and authorization of vaccines** *‘Since there is no full approval and the benefit-risk for my age group is too high from my point of view.’* Participant #1403 *‘I do not allow a gene vaccination to enter my body, which has only a conditional emergency authorization, of one knows nothing about long-term side effects. If one suffers health damage from the vaccination, no one will pay for this.’* Participant #1219 *‘Where is the scientific evidence that mercury, aluminum, and the other crud that is in vaccination cures or prevents disease? Where is the scientific proof that there are contagious viruses? The PCR test is NOT for virus detection and certainly not calibrated for the Corona virus. Health is more important to me than controversial genetic experiments!’* Participant #1037 *‘The vaccines have only an emergency approval, and there are no studies on long-term effects (is not possible after this time!)’* Participant #714 *‘I am pregnant and do not want to take any risks! For me, this vaccination is still too little documented with no long-term studies done on if it affects my unborn child and me.’* Participant #391 *‘You can also get sick from the vaccine. The vaccine can not be fully matured after such a short time, other vaccines have taken years.’* Participant #1267 *‘Untested vaccines, side effects, deaths after vaccination, blood clots, I could name 1,000 more reasons. I also do not get on an airplane that only has an emergency license. I'm not tired of living.’* Participant #100
**Fear of side effects** *‘The risk of massive side effects swept under the rug by the mainstream media: I estimate the risk to suffer health damages by Corona considerably smaller than to suffer side effects of any kind by the vaccination.’* Participant #467 *‘I want to have children someday, and I still believe that the vaccine makes you infertile.’* Participant #986 *‘I have been healthy for 40 years, and not even had a cold. I was talked into believing that the flu shot was so great and that I should definitely get the flu shot. I tried it three times - each time, 14 days after the flu shot, I got severe bronchitis and a fever over 40 degrees. No thanks, no vaccination!’* Participant #651 *‘More and more cases of vaccination side effects, some of which are extremely severe, and one almost led to death. My friend's doctor said that complications are currently more common in my age group.’* Participant #355

### Participant segmentation

Based on the Scree plot (Supplemental Figure 1) and interpretability, we decided on a maximum number of three clusters (Supplemental Figure 2). Cluster one (23%) exhibited by far the highest vaccination readiness. It showed male dominance and included participants with higher educational levels, more comorbidities, a lower fear of side effects and the desire for children (Supplemental Table 5). The first cluster might thus represent a group of people who could potentially be reached by more targeted medical information. In contrast, clusters two and three (77% in total) exhibited a lower vaccination readiness and potentially included more radical vaccination opponents: both showed lower educational levels, a higher fear of side effects, a lower trust in science, and a higher preference for political parties critical towards vaccination. While cluster two (34%) was slightly male-dominated, cluster three (43%) included more females and exhibited also the highest belief scores in ‘alternative medicine’ of all three clusters.

### Discussion and conclusion

We explored the motivational factors for COVID-19 vaccination hesitancy in Austria, a country with one of the lowest vaccination rates in Western Europe,^3^ comparatively high infection numbers, and increasing societal polarization in autumn 2021, at a point when the vaccination rate had already plateaued for a while. While the different communicative and incentive strategies tested resulted in apparent differences in the perceived attractiveness of vaccination communication, most of the strategies in the two conjoint experiments did not show significant effects on vaccination readiness. This suggests that the remaining group of unvaccinated people is not easily swayed by communication interventions alone. The most important recommendations that we can derive from the first experiment are: (1) involving healthcare providers, physicians, and scientific expertise in the campaign, (2) a fixed financial reward being preferable to a lottery and (3) easing societal restrictions, if possible, having the strongest appeal among the remaining unvaccinated. From the second experiment, further implications can be derived: (1) news coverage framing should focus on effectiveness of vaccination rather than breakthrough infections, (2) side effects should be communicated clearly and transparently using verbal and numeric information, (3) standard marketing authorization would be desirable.

Based on these results, we also expect limited impact of any intervention, including nudging strategies or even vaccine mandates, in the current situation due to the strong opposition to vaccination among a sizable subgroup of unvaccinated. Those who have not been vaccinated so far are strongly concerned about unforeseen side effects, lack trust, and show spiritual beliefs that could prevent them from getting vaccinated. Interestingly women were overrepresented among the unvaccinated individuals in our study. They showed significantly higher belief scores in paradigmatic assumptions, which individual decisions or scientific findings could scarcely influence. Likewise, strong trust in so-called ‘alternative medical methods’ such as homeopathy and the assumption that the own immune system would be sufficient in fighting COVID-19 was present in our sample.

Also, several participants considered medical conditions to be contraindications for a COVID-19 vaccination. Austria might thus have to develop strategies to improve its comparably low health literacy levels^25^^,^^26^; however, some vaccination opponents have higher science and health literacy than the average population and vaccination hesitancy in this case might be related more to the attitudes towards conventional medical methods. Otherwise, the current public discourse on the COVID-19 vaccination could also have implications on vaccination decisions against other diseases in the future. Easily accessible information campaigns for different age groups, with gender-specific information and patient organizations and health professionals as ambassadors could be considered. Beliefs in ‘alternative medicine’ would need to be scrutinized.

Furthermore, experts in bioethics have ongoing debates about the up- and downsides of monetary incentives for the COVID-19 vaccination. Among others, they warn that focusing too strongly on extrinsic motivations, like cash incentives, may ‘crowd-out’ the intrinsic motivations of citizens in the long run.^27^ Also, fairness considerations can make incentive strategies costly. Whether incentivizing citizens for medical treatment as well as to what extent lifting societal restrictions are reasonable policy options that can be brought to the table at a specific point during such a pandemic will depend on the public health situation in a given country and may have to be weighed against various ethical considerations.

Another factor contributing to vaccine hesitancy is the politicization of the pandemic, the COVID-skepticism, and the anti-vaccine mandate stance among supporters of the *‘Freedom Party of Austria (FPÖ)’* as well as the party ‘*People, Freedom, Fundamental Rights (MFG)’*. This is reflected in our sample in participants’ prospective vote choice and is accompanied by high levels of mistrust in societal institutions. Trust in general seems to be an important asset in this pandemic.^28^ A Europe-wide survey^29^ showed that Austria had the biggest loss of trust in societal institutions of all EU-27 countries between spring 2020 and 2021. Furthermore, the Eurobarometer surveys regularly show Austria among the most science-skeptic and least science-interested EU members. For example, in 2021 and compared to the EU27 average, Austrian residents perceived scientists as less intelligent, less honest, and more immoral.^30^ In the medium and long term, it would be essential to rebuild trust in all public institutions – including science – to ensure the effectiveness of evidence-based policies in times of crisis.

A limitation of our study is the cross-sectional design together with the unpredictable evolution of the Coronavirus. The omicron variant is dominant in winter 2022 in central Europe for which the currently available vaccines might offer only partial and short protection compared to the previously dominant delta variant.^31^ With omicron, a substantial proportion of the population would only have a mild or even asymptomatic disease.^32^ However, potential new virus variants in the future, decreasing immunity requiring booster vaccination and vaccination readiness for other diseases could be important fields where our results could be informative. Beyond this, we solely explored the preferences of the unvaccinated. Considerations of the impact of these measures on the already vaccinated are another important area which requires further research. Studies with other designs are needed to test the effects of vaccines and go beyond the scope of our experiment. The reliance on online access panels for recruitment in cross-sectional survey research has also limitations regarding representativeness as participating requires at least a basic level of access and skill to use technology; thus, some parts of the population remain hard to reach by online surveys.^33^ Despite that, we were nevertheless able to recruit a sample broadly matching the characteristics of the Austrian population. Other modes of data collection, e.g. telephone surveys, face their own challenges and people who are not willing to participate in a survey in general might also refuse to take part in a telephone interview. Another limitation is the self-selected nature of the participants in our study, i.e. participants chose to respond to the survey, possibly because they had more extreme or firmly held views.

## Contributors

JME, SK, JP and TS conceptualised the study; TS acquired the funding; JME, EM, JP, VR and TS curated, analysed and interpreted the data; all authors drafted and approved the manuscript.

## Funding

Vienna Science and Technology Fund (project number COV20-028).

## Declaration of Interests

JME, SK, EM, JP and VR have nothing to disclose.

TS reports grants and personal fees from AbbVie, grants and personal fees from Roche, personal fees from Sanofi, personal fees from Takeda, and personal fees from Novartis, outside the submitted work.

## Appendix Supplementary materials

Image, application 1

Image, application 2
