# Supplementary material for: Coronavirus vaccine hesitancy among unvaccinated Austrians: Assessing underlying motivations and the effectiveness of interventions based on a cross-sectional survey with two embedded conjoint experiments
Source: Lancet Reg Health Eur. 2022 Apr 22;17:100389. doi: 10.1016/j.lanepe.2022.100389 (PMC9023089; doi:10.1016/j.lanepe.2022.100389)
Supplement: Supplementary file 1 [file mmc1.docx]

**Appendix with supplemental information**

**Supplemental File 1. Details on the survey.** The questions included the pre-defined stratification criteria, vaccination status, whether the participants were currently ill or recovered from COVID-19, how often they underwent testing for COVID-19, whether they had contact with people who were seriously ill from COVID-19, how they would estimate the impact of the restrictive measures to mitigate the virus transmission from an economic perspective, how much trust they had in official institutions, how they would estimate their health status, household wealth and size, desire to have children in the future, whether they were currently pregnant (for females only), and which political party they would vote for if national council elections would take place on the coming weekend.

We also included an open question on the reason for not being vaccinated and asked the participants to indicate their level of agreement with statements on side effects of vaccination and health status. We used either numerical rating scales, such as 11-point scales from 0 to 10, to ask about levels of trust or Likert-type scales where participants could indicate their agreement with certain statements.

**Supplemental File 2. Sample size calculation.** We calculated a minimum sample size of 1,399 for the conjoint experiment based on the size of the Austrian population, an assumed incidence rate of an elevated vaccination readiness of approximately 35% among the unvaccinated, desired confidence levels of 95%, and error margins of 2.5%. Previous population-representative surveys also had comparable sample sizes of about 1,500 Austrian residents.^1^

*1. Kittel B, Kritzinger S, Boomgaarden H, et al. The Austrian Corona Panel Project: monitoring individual and societal dynamics amidst the COVID-19 crisis. European Political Science 2021; 20(2): 318-44.*

**Supplemental File 3. Details on missing values.** Participants had to answer each question to get to the next one. However, they could tick the answer options ‘I do not want to answer’ or ‘I do not know’, where appropriate for the type of question. We show the number of the participants who chose one of these two options in Tables 1 and 2, where applicable, as well as in Figure 2 (the grey areas on top of the bars). In the results section, we also report the number of the undecided regarding the prospective vote choice.

To answer the open question was not mandatory and we received answers from 391 participants (25%) on potential reasons for not being vaccinated. From the cluster analysis, we had to exclude 687 participants (45%) because they ticked in at least once in one of the target variables ‘I do not know’ or ‘I do not want to answer’.

**Supplemental Table 1. Details on experiment 1.** This table shows the attributes and levels of Experiment 1. For the introduction, we used the following text: ‘In the following, we show you various calls for Corona vaccination. In each case, these are made-up framework conditions under which calls for Corona vaccination are made.’

| **Attributes** | **Levels** |
| --- | --- |
| Call for vaccination | - Many people have been vaccinated. - Many people have been vaccinated to help protect their fellow human beings from a serious course of the disease. - Many people have been vaccinated to protect themselves from a serious course of disease. - Many people have been vaccinated so that life can return to the way it was before the pandemic. |
| Recommended by | - The Federal Government - Physicians - Prominent person: most likeable celebrity from a previous question in case of a tie or all unknown or no indication: <random selection>. |
| Incentives | - The vaccination is free of charge. - The vaccination is free of charge and you will receive 100 euros in compensation. - For every 200 newly vaccinated people, 20,000 euros will be raffled. All vaccinated people are allowed to participate in the raffle. |
| Rules | - 3G rule - access only for vaccinated, recovered, and tested persons - 2G rule - access only for vaccinated and recovered persons - All rules are cancelled |

Levels were randomly assigned and repeatedly recombined. This resulted in 108 different combinations for experiment 1.

For each pair of vignettes, we asked the participants which of these two calls would appeal to them more; in case neither would appeal to them nor both would equally, they should decide spontaneously. We then asked the participants to compare and rate both vignettes separately regarding their personal willingness to be vaccinated on a scale from 0 (‘Would definitely not get vaccinated’) to 10 (‘Would definitely get vaccinated’).

**Supplemental Table 2. Details on experiment 2.** This table shows the attributes and levels of Experiment 2. For the introduction, we used the following text: ‘Below, we show you some made-up media reports about a hypothetical vaccine against a new virus that is highly contagious.’

| **Attributes** | **Levels** |
| --- | --- |
| Effectiveness and vaccination breakthroughs | - The vaccine has a 90% efficacy in preventing severe disease progression. 90% efficacy means 90% fewer hospitalized due to viral infection among the vaccinated compared to the unvaccinated. In other words, there is one vaccinated person for every 10 hospitalized unvaccinated persons. - 60% of the population in a country is vaccinated. In recent months, 500,000 people became infected. Of these, 4,189 vaccinated people and 43,310 unvaccinated people required hospital treatment for the viral infection. - Health authorities report that 4,189 people had to be hospitalized for the viral infection even though they were vaccinated. |
| Risk communication | - The vaccine, like any other vaccine, has side effects. - The vaccine has mild and severe side effects: Mild side effects, such as flu-like symptoms, are common. Severe side effects that require hospital treatment are extremely rare. - The vaccine has mild and severe side effects: Mild side effects, such as flu-like symptoms, occur frequently (in 4 out of 10 people). Severe side effects requiring hospital treatment are extremely rare (in 4 out of 1,000,000 people). - The vaccine has mild and severe side effects: Mild side effects, such as flu-like symptoms, are common (in 4 out of 10 people). Severe side effects requiring hospital treatment are extremely rare (in 4 out of 1,000,000 people).  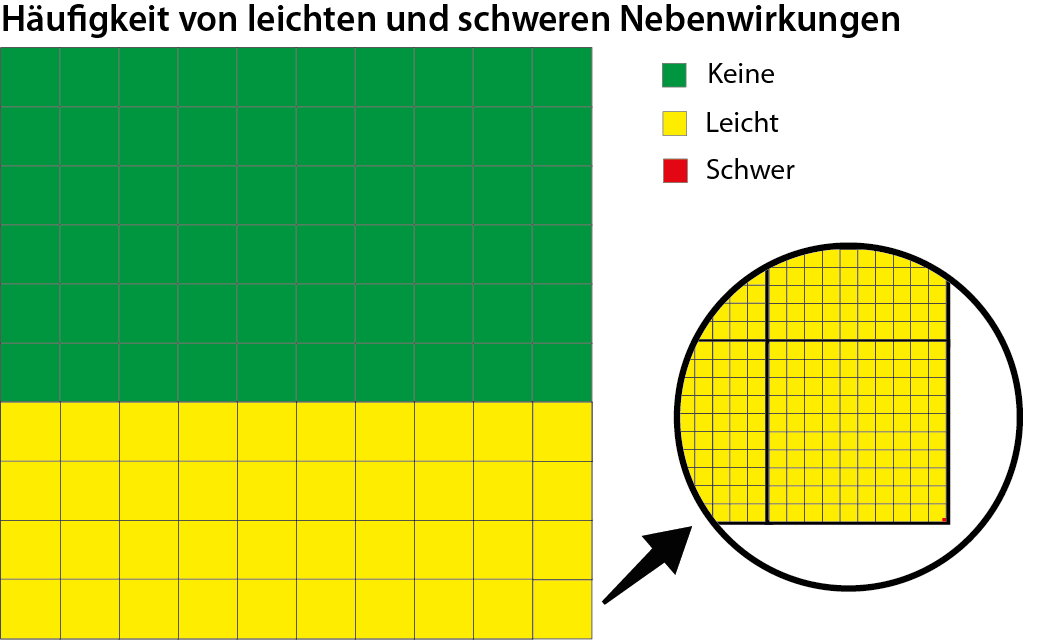 |
| Admission procedure | - The vaccine has undergone a standard approval procedure by Austrian authorities. - The vaccine has undergone a standard approval procedure by European authorities. - The vaccine has undergone an accelerated procedure (conditional marketing authorization) by European authorities. |

Levels were randomly assigned and repeatedly recombined. This resulted in 36 different combinations for experiment 2.

For each pair of vignettes, we asked the participants which of these two calls would appeal to them more; in case neither would appeal to them nor both would equally, they should decide spontaneously. We then asked the participants to compare and rate both vignettes separately regarding their personal willingness to be vaccinated on a scale from 0 (‘Would definitely not get vaccinated’) to 10 (‘Would definitely get vaccinated’).

**Supplemental Table 3.** **Average Marginal Component Effects (AMCEs) from the ratings of vaccination readiness and the preferred choices regarding case vignette’s appeal from experiment 1.** Significant p-values are marked in bold; the Bonferroni corrected significance level is 0.003. ‘Lower’ and ‘Upper’ refers to the 95% CIs.

| **Feature** | **Level** | **Estimate** | **Std. error** | **z** | **p-value** | **Lower** | **Upper** |
| --- | --- | --- | --- | --- | --- | --- | --- |
| **Choices** |  |  |  |  |  |  |  |
| Call | No specific reason given | 0 | **-** |  |  |  |  |
|  | To protect oneself | -0.002 | 0.017 | -0.093 | 0.926 | -0.036 | 0.033 |
|  | To protect others | -0.010 | 0.017 | -0.583 | 0.560 | -0.044 | 0.024 |
|  | To return to normality | -0.002 | 0.017 | -0.106 | 0.915 | -0.035 | 0.032 |
| Reco | Celebrity | 0 |  |  |  |  |  |
|  | Federal government | 0.000 | 0.016 | -0.006 | 0.995 | -0.031 | 0.030 |
|  | Physician | 0.120 | 0.016 | 7.669 | **<0.001** | 0.089 | 0.150 |
| Incen | Lottery | 0 |  |  |  |  |  |
|  | Monetary gift | 0.105 | 0.015 | 6.821 | **<0.001** | 0.075 | 0.135 |
|  | Only the vaccination being free of charge | 0.123 | 0.015 | 7.991 | **<0.001** | 0.093 | 0.154 |
| Rule | 2G Rule | 0 |  |  |  |  |  |
|  | 3G Rule | 0.205 | 0.015 | 13.670 | **<0.001** | 0.175 | 0.234 |
|  | Lifting all restrictions | 0.303 | 0.015 | 19.839 | **<0.001** | 0.273 | 0.333 |
| **Ratings** |  |  |  |  |  |  |  |
| Call | No specific reason given | 0 | - | - | - | - | - |
|  | To protect oneself | 0.113 | 0.098 | 1.153 | 0.249 | -0.079 | 0.306 |
|  | To protect others | 0.073 | 0.088 | 0.826 | 0.409 | -0.100 | 0.246 |
|  | To return to normality | 0.055 | 0.093 | 0.599 | 0.549 | -0.126 | 0.237 |
| Reco | Celebrity | 0 | - | - | - | - | - |
|  | Federal government | 0.048 | 0.082 | 0.592 | 0.554 | -0.112 | 0.209 |
|  | Physician | 0.143 | 0.081 | 1.764 | 0.078 | -0.016 | 0.302 |
| Incen | Lottery | 0 | - | - | - | - | - |
|  | Monetary gift | 0.177 | 0.080 | 2.220 | 0.026 | 0.021 | 0.334 |
|  | Only the vaccination being free of charge | 0.028 | 0.077 | 0.368 | 0.713 | -0.123 | 0.180 |
| Rule | 2G Rule | 0 | - | - | - | - | - |
|  | 3G Rule | 0.162 | 0.078 | 2.075 | 0.038 | 0.009 | 0.315 |
|  | Lifting all restrictions | 0.321 | 0.079 | 4.066 | **<0.001** | 0.166 | 0.476 |

**Supplemental Table 4.** **Average Marginal Component Effects (AMCEs) from the ratings of vaccination readiness and the preferred choices regarding case vignette’s appeal from experiment 2.** Significant p-values are marked in bold. The Bonferroni corrected significance level is 0.004. ‘Lower’ and ‘Upper’ refer to the 95% CIs.

| **Feature** | **Level** | **Estimate** | **Std.error** | **z** | **p** | **Lower** | **Upper** |
| --- | --- | --- | --- | --- | --- | --- | --- |
| **Choices** |  |  |  |  |  |  |  |
| Eff | Absolute numbers of breakthrough infections | 0 |  |  |  |  |  |
|  | 90% effectiveness | 0.058 | 0.016 | 3.603 | **<0.001** | 0.026 | 0.089 |
|  | Rel. and abs. frequ. | -0.006 | 0.016 | -0.406 | 0.685 | -0.037 | 0.025 |
| Risk | Vague verbal info on side effects that can occur | 0 |  |  |  |  |  |
|  | Vague verbal info regarding frequ. | 0.016 | 0.018 | 0.860 | 0.390 | -0.020 | 0.052 |
|  | Verbal and numeric info on frequ. | 0.071 | 0.019 | 3.823 | **<0.001** | 0.035 | 0.108 |
|  | Verbal and numeric info on frequ. + infographic | 0.045 | 0.019 | 2.425 | 0.015 | 0.009 | 0.082 |
| Admission | Conditional market authorisation EU level | 0 |  |  |  |  |  |
|  | Standard market author. EU | 0.168 | 0.016 | 10.640 | **<0.001** | 0.137 | 0.200 |
|  | Standard market author. AT | 0.184 | 0.016 | 11.370 | **<0.001** | 0.152 | 0.216 |
| **Ratings** |  |  |  |  |  |  |  |
| Eff | Absolute numbers of breakthrough infections | 0 |  |  |  |  |  |
|  | 90% effectiveness | 0.102 | 0.074 | 1.382 | 0.167 | -0.043 | 0.248 |
|  | Rel. and abs. frequ. | 0.031 | 0.076 | 0.413 | 0.679 | -0.117 | 0.179 |
| Risk | Vague verbal info on side effects that can occur | 0 |  |  |  |  |  |
|  | Vague verbal info regarding frequ. | 0.036 | 0.087 | 0.417 | 0.676 | -0.135 | 0.208 |
|  | Verbal and numeric info on frequ. | 0.049 | 0.089 | 0.547 | 0.584 | -0.126 | 0.224 |
|  | Verbal and numeric info on frequ. + infographic | 0.013 | 0.089 | 0.142 | 0.887 | -0.161 | 0.186 |
| Admission | Conditional market authorisation EU level | 0 |  |  |  |  |  |
|  | Standard market author. EU | 0.362 | 0.073 | 4.976 | **<0.001** | 0.219 | 0.505 |
|  | Standard market author. AT | 0.295 | 0.071 | 4.138 | **<0.001** | 0.155 | 0.435 |

**Supplemental Figure 1. Scree plot to determine the number of clusters.** The within-cluster variance is reaching an acceptably low level at the 3^rd^ point, as the graph slightly flattens there. We also considered interpretability to avoid too much overlap between the clusters.


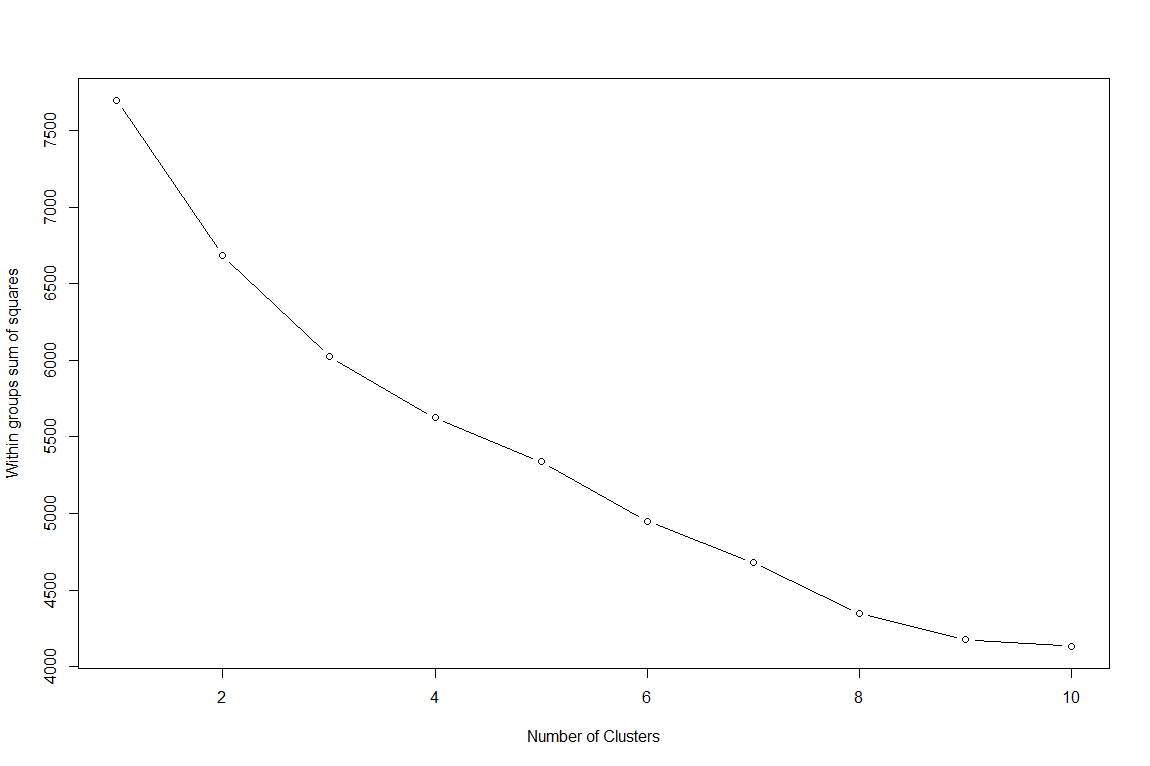


**Supplemental Figure 2.** **Cluster means and sizes.** Higher scores indicate a higher vaccination readiness *(Vacc. R.)*, female gender *(Gender)*, more years of education *(Edu.)*, more comorbidities *(Comorb.)*, a higher belief in homeopathy *(Homeop.)*, a higher preference for political parties critical towards vaccination *(Polit. Pref.)*, a higher trust in science *(Trust. Scie.)*, a higher fear of unforeseen side effects *(Side Eff.)* and a desire to have own children in the future *(Des. Child.)*. We thus overall could identify three kernels in our sample which might require different intervention strategies: (1) a group of people with comorbidities or in the child-bearing age with potentially little access to valid information, (2) a group of male populist sympathizers, (3) a female-dominated group of people with equal political preferences as in cluster 2 who, in addition, strongly believe in paradigmatic assumptions and alternative medicines.


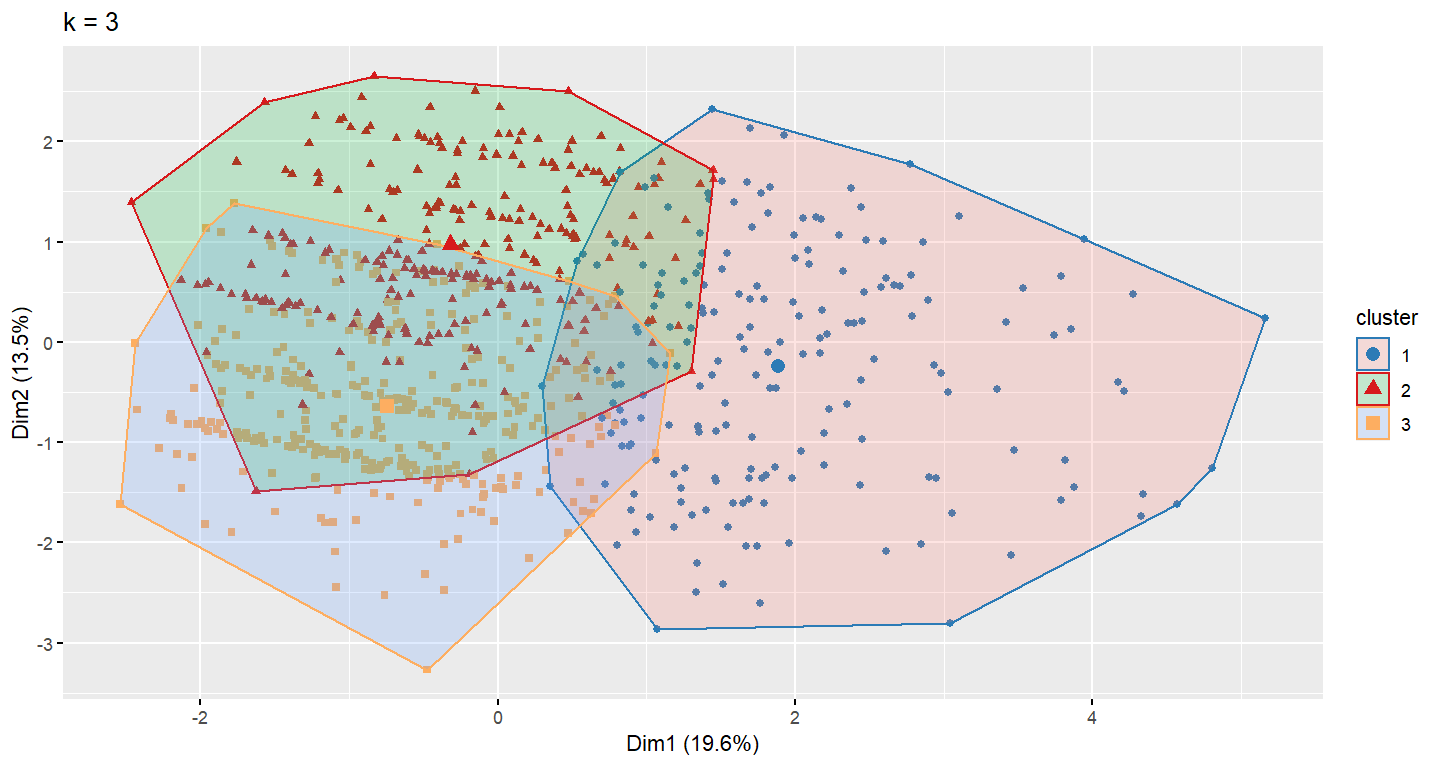


**Supplemental Table 5. Characteristics of clusters.**

| # | n | Vacc. R. | Gender | Edu. | Comorb. | Homeop. | Polit. Pref. | Trust. Scie. | Side Eff. | Des. Child. |
| --- | --- | --- | --- | --- | --- | --- | --- | --- | --- | --- |
| 1 | 195(23%) | **1.327** | -0.116 | **0.188** | **0.199** | -0.180 | -0.522 | **0.626** | **-1.033** | **0.504** |
| 2 | 293(34%) | -0.365 | **-1.082** | -0.025 | -0.150 | -0.194 | 0.148 | -0.190 | 0.283 | -0.150 |
| 3 | 368(43%) | -0.413 | 0.923 | -0.080 | 0.014 | **0.249** | **0.159** | -0.180 | 0.322 | -0.148 |

*Notes*: Entries are normalized mean scores; the highest absolut value in each category is marked in bold. Birth year performed just opposite the desire for own children and was, therefore, not included in the final segmentation model.
