# Supplementary material for: Coronavirus vaccine hesitancy among unvaccinated Austrians: Assessing underlying motivations and the effectiveness of interventions based on a cross-sectional survey with two embedded conjoint experiments
Source: Lancet Reg Health Eur. 2022 Apr 22;17:100389. doi: 10.1016/j.lanepe.2022.100389 (PMC9023089; doi:10.1016/j.lanepe.2022.100389)
Supplement: Supplementary file 2 [file mmc2.docx]

**Caption for supplementary material**

This document includes a list of captions for each Supplementary file as they should appear online.

| **File** | **Table/Figure** | **Caption for online appearance** |
| --- | --- | --- |
| Appendix with supplemental information |  | Appendix |
|  | Supplemental File 1. | Supplemental File 1. |
|  | Supplemental File 2. | Supplemental File 2. |
|  | Supplemental File 3. | Supplemental File 3. |
|  | Supplemental Table 1. | Supplemental Table 1. |
|  | Supplemental Table 2. | Supplemental Table 2. |
|  | Supplemental Table 3. | Supplemental Table 3. |
|  | Supplemental Table 4. | Supplemental Table 4. |
|  | Supplemental Figure 1. | Supplemental Figure 1. |
|  | Supplemental Figure 2. | Supplemental Figure 2. |
|  | Supplemental Table 5. | Supplemental Table 5. |
| Supplemental_File_4_STROBE-checklist-v4-combined_2022_02_02 |  | Supplemental File 4. STROBE Checklist |
